# VEGFA’s distal enhancer regulates its alternative splicing in CML

**DOI:** 10.1093/narcan/zcab029

**Published:** 2021-07-13

**Authors:** Sara Dahan, Aveksha Sharma, Klil Cohen, Mai Baker, Nadeen Taqatqa, Mercedes Bentata, Eden Engal, Ahmad Siam, Gillian Kay, Yotam Drier, Shlomo Elias, Maayan Salton

**Affiliations:** Department of Biochemistry and Molecular Biology, The Institute for Medical Research Israel–Canada, Hebrew University–Hadassah Medical School, Jerusalem 91120, Israel; Department of Biochemistry and Molecular Biology, The Institute for Medical Research Israel–Canada, Hebrew University–Hadassah Medical School, Jerusalem 91120, Israel; Department of Biochemistry and Molecular Biology, The Institute for Medical Research Israel–Canada, Hebrew University–Hadassah Medical School, Jerusalem 91120, Israel; Department of Biochemistry and Molecular Biology, The Institute for Medical Research Israel–Canada, Hebrew University–Hadassah Medical School, Jerusalem 91120, Israel; Department of Biochemistry and Molecular Biology, The Institute for Medical Research Israel–Canada, Hebrew University–Hadassah Medical School, Jerusalem 91120, Israel; Department of Biochemistry and Molecular Biology, The Institute for Medical Research Israel–Canada, Hebrew University–Hadassah Medical School, Jerusalem 91120, Israel; Department of Biochemistry and Molecular Biology, The Institute for Medical Research Israel–Canada, Hebrew University–Hadassah Medical School, Jerusalem 91120, Israel; Department of Biochemistry and Molecular Biology, The Institute for Medical Research Israel–Canada, Hebrew University–Hadassah Medical School, Jerusalem 91120, Israel; Department of Biochemistry and Molecular Biology, The Institute for Medical Research Israel–Canada, Hebrew University–Hadassah Medical School, Jerusalem 91120, Israel; The Lautenberg Center for Immunology and Cancer Research, IMRIC, Faculty of Medicine, The Hebrew University–Hadassah Medical School, Jerusalem 91120, Israel; Department of Hematology, Hadassah–Hebrew University Medical Center, Jerusalem 91120, Israel; Department of Biochemistry and Molecular Biology, The Institute for Medical Research Israel–Canada, Hebrew University–Hadassah Medical School, Jerusalem 91120, Israel

## Abstract

Enhancer demethylation in leukemia has been shown to lead to overexpression of genes which promote cancer characteristics. The vascular endothelial growth factor A (VEGFA) enhancer, located 157 Kb downstream of its promoter, is demethylated in chronic myeloid leukemia (CML). VEGFA has several alternative splicing isoforms with different roles in cancer progression. Since transcription and splicing are coupled, we wondered whether VEGFA enhancer activity can also regulate the gene’s alternative splicing to contribute to the pathology of CML. Our results show that mutating the VEGFA +157 enhancer promotes exclusion of exons 6a and 7 and activating the enhancer by tethering a chromatin activator has the opposite effect. In line with these results, CML patients present with high expression of +157 eRNA and inclusion of VEGFA exons 6a and 7. In addition, our results show that the positive regulator of RNAPII transcription elongation, CCNT2, binds VEGFA’s promoter and enhancer, and its silencing promotes exclusion of exons 6a and 7 as it slows down RNAPII elongation rate. Thus our results suggest that VEGFA’s +157 enhancer regulates its alternative splicing by increasing RNAPII elongation rate via CCNT2. Our work demonstrates for the first time a connection between an endogenous enhancer and alternative splicing regulation of its target gene.

## Graphical Abstract

**Graphical Abstract:**
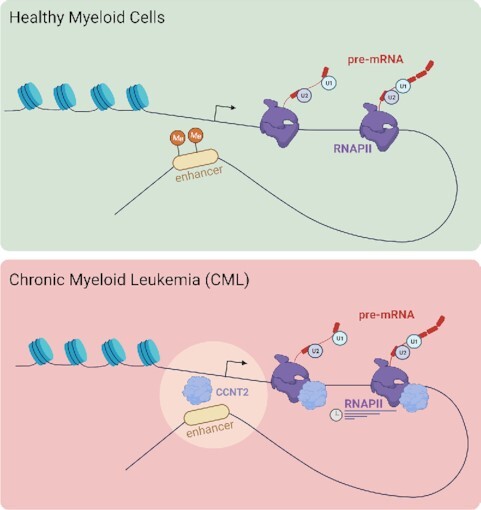
Enhancing alternative splicing.

## INTRODUCTION

Transcriptional enhancers are major regulators of tissue-specific gene expression. During cellular differentiation, enhancers that control the expression of genes involved in lineage specification become active, while others are deactivated. The disruption of enhancer activity, through genetic or epigenetic alterations, can impact cell-type-specific functions and promote tumorigenesis of various cancers (1). Thus, the maintenance of cell-type-specific enhancer activation is critical to prevent disease.

Enhancer DNA methylation was shown to play an important part in maintaining and modifying enhancer activity during differentiation (2). Vascular endothelial growth factor A (VEGFA) enhancer, located 157 Kb downstream to the gene’s promoter (+157), is active in embryonic stem cells, but during hematopoiesis it becomes methylated and inactive (2). In chronic myeloid leukemia (CML), the enhancer is demethylated and VEGFA is overexpressed (2). VEGFA overexpression has been shown to be correlated with a range of tumor types (3–5) and to promote growth and survival of vascular endothelial cells. While vascularization is critical in solid tumors to allow for oxygen and nutrient flux (6), it has been shown to be present in hematological malignancies as well (7,8). Interestingly, VEGFA pre-mRNA alternative splicing changes in many types of cancer (9–12) and alternative splicing in general are known to be affected by RNA polymerase II (RNAPII) activity (13).

CML is triggered by a Philadelphia chromosome encoding the BCR-ABL oncogenic fusion protein with constitutive and aberrant tyrosine kinase activity (14). The introduction of tyrosine kinase inhibitors (TKIs) revolutionized the management of CML and patients’ life expectancy is now approaching normality (15). Regardless, some CML patients do not respond to TKIs and progress to acute leukemia (16). BCR-ABL was shown to induce the expression of hypoxia-inducible factor-1 (HIF-1), a transcription factor binding to the VEGFA promoter and enhancer regions (17,18). In addition, drugs targeting BCR-ABL reduce VEGFA expression (19,20). In turn VEGFA overexpression is associated with the clinical characteristics of CML (21). High levels of VEGFA were recorded in the blood as well as the bone marrow of CML patients and were correlated with high vascularization and proliferation leading to the pathology of CML (22). VEGFA alternative splicing isoforms have different roles in vascularization and cancer progression, but their expression in CML has not been studied.

In the process of alternative splicing, different protein isoforms can arise from the same gene. These proteins can have different functions that could affect the cellular state. In this context, many proteins as well as small non-coding RNAs take part in the tight regulation of the alternative splicing process. Changes to alternative splicing are common in many diseases, including cancer; alternative splicing misregulation in cancer cells promotes the formation of cancer-driving isoforms (23). These isoforms have been shown to be involved in each of the hallmarks of cancer (reviewed in 24); and splicing inhibitors can serve as a therapy for many types of cancers (25,26).

A strong connection between transcription and splicing has been known for 30 years (27). Manipulating a gene’s promoter or positioning a synthetic enhancer next to a gene can alter alternative splicing (27–29). In addition, slow kinetics of RNAPII elongation promotes exon inclusion; the included exon is the first to transcribe and consequently the first to be identified by the spliceosome (30). In detail, slowing transcription elongation rate can change the time allowed for the spliceosome to be recruited to a specific splice site and so prevent it from being identified, thereby promoting exon inclusion. On the contrary, RNAPII fast kinetics promotes exon exclusion as it exposes additional splice sites (30). The opposite effect can also be observed whereby slow RNAPII kinetics promotes exon exclusion. This effect was attributed to an inhibitory splicing factor that gains a binding opportunity when RNAPII is slow to transcribe (31). This connection between transcription and alternative splicing suggests that a change in transcription in cancer cells can lead to a change in gene expression as well as alternative splicing.

**Figure 1.**
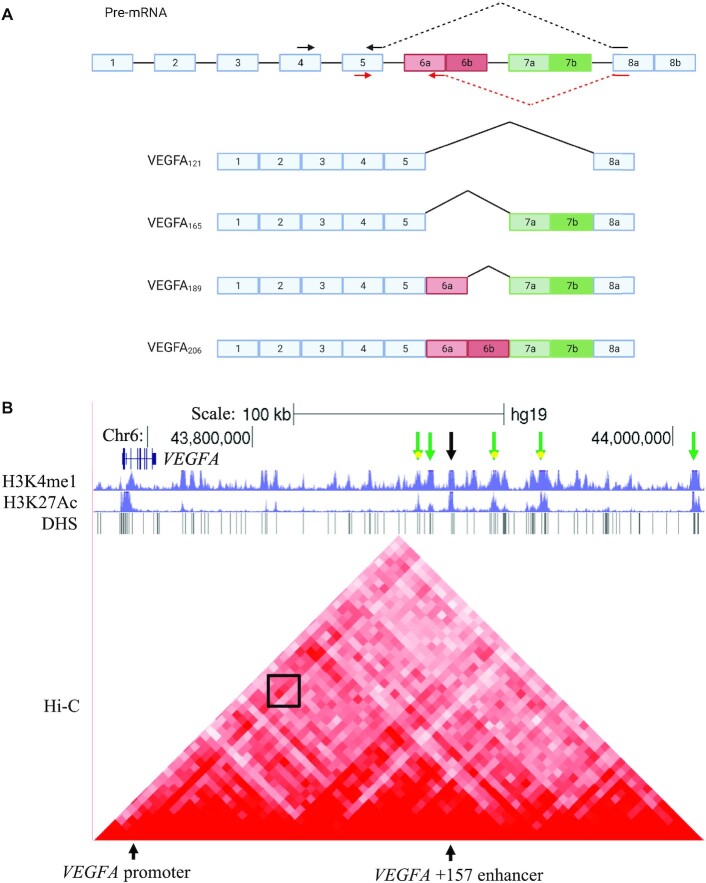
VEGFA isoforms and +157 enhancer region. (**A**) Schematic representation of the VEGFA gene and four of its known isoforms. Rectangles: exons, black lines: introns. Arrows in black indicate primers for isoform VEGFA_121_ and arrows in red for VEGFA_189_. (**B**) ChIP-seq tracks for H3K27ac and H3K4me1 including DHS and Hi-C data (5 kb resolution) at the VEGFA locus in K562 cells. VEGFA +157 enhancer is depicted with a black arrow and enriched for H3K27ac and H3K4me1. The Hi-C data suggest a sub-TAD between VEGFA +157 enhancer and the promoter, marked with a black box. Green arrows depict additional enhancers with sub-TADs to VEGFA promoter.

Our results demonstrate that alternative splicing of VEGFA is co-regulated with its expression by its +157 enhancer. VEGFA’s major splicing isoforms are VEGFA_121_, VEGFA_165_, VEGFA_189_ and VEGFA_206_; these isoforms are generated by alternative splicing of 6a/b and 7a/b with exons 1–5 and exon 8a as constitutive exons (3) (Figure 1A). The isoforms' relative abundance in cancer is correlated with pathogenesis but their functional roles are poorly characterized (9–12). To learn about the connection of the VEGFA enhancer to its alternative splicing, we used the CML cell line, K562, mutated at the VEGFA enhancer using the CRISPR/Cas9 genome-editing system. It has previously been shown that mutations at this site reduce the total mRNA amount of VEGFA (2) and we further found that it regulates its alternative splicing to promote exclusion of VEGFA exons 6a and 7, which translates to VEGFA_121_. Activating the enhancer using CRISPR/dCas9 to tether p300 gave the opposite result. In line with this result, CML patients show high expression of +157 eRNA and inclusion of VEGFA exons 6a and 7, which translates to VEGFA_189_. To study the mechanism of enhancer regulation of alternative splicing, we altered RNAPII transcription elongation kinetics and monitored alternative splicing. We found that similar to mutations at the +157 enhancer, slowing down RNAPII elongation promotes exclusion of VEGFA exons 6a and 7. Finally we identified PML and CCNT2 as chromatin factors binding to VEGFA promoter and +157 enhancer and as regulators of its alternative splicing. CCNT2 is a known positive regulator of RNAPII and indeed our results demonstrate that CCNT2 mediates RNAPII elongation kinetics caused by the +157 enhancer, which regulates VEGFA alternative splicing. Ultimately, our work identifies for the first time a connection between an endogenous enhancer and alternative splicing regulation of its target gene.

## MATERIALS AND METHODS

### Ethics approval

The Hadassah Medical Organization Institutional Board approved gathering samples for this project (0364–17-HMO), based on the Helsinki Declaration. This authorization is specifically applicable to the experiments reported in the paper.

### Cell lines and plasmids

HEK293T (ATCC Number: CRL-3216) and K562 (ATCC Number: CCL-243) cells were grown in Dulbecco’s modified Eagle’s medium (DMEM) and RPMI-1640, respectively, supplemented with 10% fetal bovine serum. Cell lines were maintained at 37°C and 5% CO_2_ atmosphere. Cells were transfected with nucleofection (Amaxa). After 30 h in culture, plasmid-transfected cells were used for experimentation.

pcDNA-dCas9-p300 core plasmids (D1399Y; plasmid #61358 and plasmid #61357) were purchased from Addgene. pSPgRNA (Addgene, plasmid #47108) was used as the gRNA plasmid. The oligonucleotides containing the target sequences were hybridized, phosphorylated and cloned into the plasmid using BbsI sites. The target sequences are provided in Supplementary Table S3.

### RNAi

OnTarget Plus SMART pool of four siRNA oligomers per gene against PML, CCNT2 and MAZ were purchased from Sigma. Cells were grown to 20–50% confluence and transfected with siRNA using nucleofection (Amaxa). After 72 h in culture, siRNA transfected cells were used for experimentation.

### qRT-PCR

RNA was isolated from cells using the GENEzol TriRNA Pure Kit (GeneAid). cDNA synthesis was carried out with the Quanta cDNA Reverse Transcription Kit (QuantaBio). qPCR was performed with the iTaq Supermix (BioRad) on the BioRad iCycler. For *BCR-ABL* quantification, mononuclear cells derived from peripheral blood of CML patients were isolated on Ficoll-Hypaque gradient and total RNA was isolated. Real-time for *BCR-ABL* quantification was performed using ipsogen® BCR-ABL1 Mbcr IS-MMR (Qiagen). The comparative *C*t method was employed to quantify transcripts, and delta *C*t was measured in triplicate. Primers used in this study are provided in Supplementary Table S3.

### DRB treatment

For elongation rate assessment, 5,6-dichlorobenzimidazole 1-β-D-ribofuranoside (DRB; Sigma-Aldrich) dissolved in DMSO (final concentration 100 μM) or DMSO alone (0.1% [v/v]) as vehicle control was added to the medium. After incubation of 3 h, medium was removed and the cells were incubated in fresh medium for the indicated time points.

### Immunoblotting

For immunoblotting, cells were harvested and lysed with RIPA lysis buffer, and 20 μg/μl of the extracts were run on a 4–12% Bis-Tris gel and transferred onto a nitrocellulose membrane. Antibodies used for immunoblotting were PML (sc-377390), CCNT2 (sc-81243) and MAZ (sc-28745) from Santa Cruz Biotechnology.

### ChIP

Approximately 2 × 10^6^ cells per sample were cross-linked for 15 min in 1% formaldehyde at RT. Cells were washed twice with cold PBS and lysed with lysis buffer (0.5% sodium dodecyl sulfate [SDS], 10 mM ethylenediaminetetraacetic acid [EDTA], 50 mM Tris–HCl, pH 8 and 1×protease inhibitor cocktail). DNA was sonicated in an ultrasonic bath (Qsonica, Q800R2 Sonicator) to an average length of 200–1000 bp. Supernatants were immunoprecipitated overnight with 40 μl of precoated anti-IgG magnetic beads (goat anti-rabbit IgG magnetic beads, NEB) previously incubated with the antibody of interest for 6 h at 4°C. The antibodies used were rabbit anti-H3ac, acetyl K9 + K14 + K18 + K23 + K27 (Abcam, cat. no. ab47915) and rabbit anti-histone H3 (Abcam, cat. no. ab1791). Beads were washed sequentially for 5 min each in low-salt (20 mM Tris–HCl pH 8, 150 mM NaCl, 2 mM EDTA, 1% Triton X-100, 0.1% SDS), high-salt (20 mM Tris–HCl pH 8, 500 mM NaCl, 2 mM EDTA, 1% Triton X-100, 0.1% SDS) and LiCl buffer (10 mM Tris pH 8.0, 1 mM EDTA, 250 mM LiCl, 1% NP-40, 1% Nadeoxycholate) and in Tris-EDTA (TE) buffer. Beads were eluted in 1% SDS and 100 mM NaHCO_3_ buffer for 15 min at 65°C and cross-linking was reversed for 6 h after addition of NaCl to a final concentration of 200 mM and sequentially treated with 20 μg proteinase K. DNA was extracted using magnetic beads (Beckman Coulter, Agencourt AMPure XP, cat. no. A63881). Immunoprecipitated DNA (2 out of 50 μl) and serial dilutions of the 10% input DNA (1:5, 1:25, 1:125 and 1:625) were analyzed by SYBR-Green real-time qPCR. ChIP-qPCR data were analyzed relative to input to include normalization for both background levels and the amount of input chromatin to be used in ChIP. The primer sequences used are listed in Supplementary Table S3.

### CPT treatment

To impede the dynamics of transcribing RNAPII, cells were treated with camptothecin (CPT, Sigma) to a final concentration of 6 μM for 6 h.

### Cell proliferation

Suspensions of K562 cells in logarithmic growth at a cell density of 2 × 10^4^/ml were seeded in T25 flasks. Cell cultures were seeded in triplicates, and were incubated for the time indicated. Cells were counted and the mean value was calculated for each time point relative to day 1.

## RESULTS

### VEGFA +157 enhancer is marked by H3K4me1, H3K27ac and DNase-seq and loops to its promoter

Enhancers are marked with specific histone modifications, including monomethylation of histone H3 on Lys 4 (H3K4me1) and acetylation of histone H3 on Lys 27 (H3K27ac). They are also associated with regions of nucleosome depletion, exhibiting high sensitivity to DNA nucleases such as the DNase I, forming DNase hypersensitivity sites (DHS). We analyzed histone modification and DHS data in K562 cells (ENCODE Project Consortium (32)) and found that indeed the *VEGFA* +157 enhancer is marked by H3K4me1 and H3K27ac and exhibits multiple DHS sites. Moreover, we analyzed K562 Hi-C chromosome conformation capture data (32) and identified a stripe architecture (33) at the VEGFA promoter, with marked interaction between the promoter and the *VEGFA* +157 enhancer, suggesting they frequently interact (Figure 1B). Hi-C data for this region also indicate that VEGFA promoter and +157 enhancer can be found in the same TAD (Supplementary Figure S1A). In addition, we identified five additional putative VEGFA enhancers with similar characteristics (Figure 1B). We chose to begin our investigation with VEGFA +157 enhancer as it is the only experimentally verified enhancer (2).

### Mutations in VEGFA +157 enhancer promote exclusion of VEGFA exons 6a and 7

**Figure 2.**
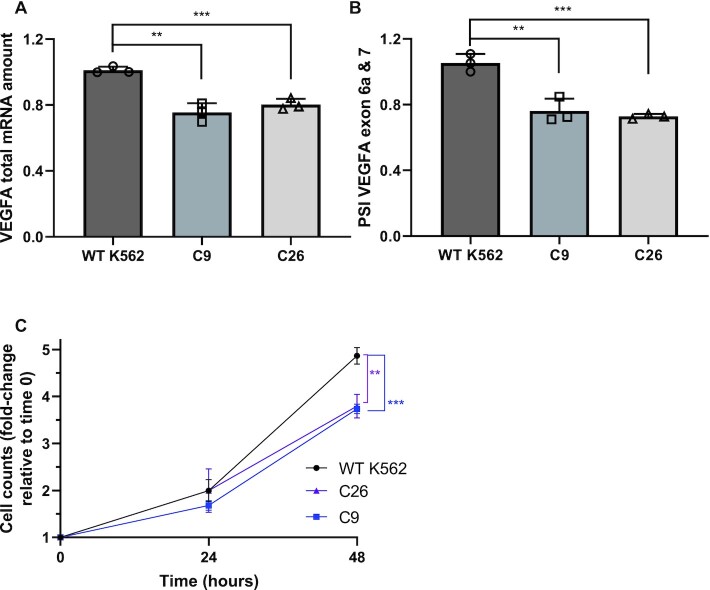
Mutating the VEGFA +157 enhancer leads to VEGFA exons 6a and 7 exclusion and to slower growth of these cells. (**A** and**B**) RNA was extracted from WT K562 cells and enhancer mutated clones 9 and 26 and analyzed by real-time PCR for total mRNA amount of VEGFA relative to *CycloA* and *hTBP* reference genes (A) and for VEGFA_121_ and VEGFA_189_ relative to VEGFA total mRNA amount. PSI was calculated by VEGFA_189_ relative to VEGFA_121_ (B). Plots represents the mean of three independent experiments and ± SD (*P< 0.05; **P< 0.01, ***P< 0.001). (**C**) WT K562 cells and enhancer mutated C9 and C26 were seeded and counted every day. Plot represents the mean of three independent experiments done with three replicates and ± SD (*P< 0.05, Student’s *t* test).

To check whether VEGFA’s enhancer regulates VEGFA’s alternative splicing, we used the CML cell line, K562, with mutations in VEGFA + 157 enhancer (2). We used two clones, 9 and 26, generated using the CRISPR/Cas9 genome-editing system. Clone 9 has a single nucleotide insertion at the three chromosome 6 copies in this cell line, while clone 26 has various deletions (Supplementary Figure S1B). We began by measuring VEGFA total mRNA in the parental K562 cell line and in clones 9 and 26. As was described before (2), we detected a 20–30% reduction in VEGFA total mRNA following the impairment of the +157 enhancer (Figure 2A). To check the transcription level of VEGFA in these cells, we used primers spanning the exon-intron junction to measure nascent pre-mRNA. The level of transcription had similar trend to VEGFA total mRNA amount indicating lower transcription rate and not a change in mature mRNA half-life (Supplementary Figure S2). Furthermore, we detected exclusion of VEGFA exons 6a and 7 in mutated +157 enhancer cells (Figure 2B). Our results suggest that VEGFA +157 enhancer promotes inclusion of exons 6a and 7 giving rise to VEGFA_189_ and a reduction in VEGFA_121_.

### +157 enhancer is important for cell proliferation

We have previously shown that hypoxia and induction of VEGFA expression result in inclusion of exons 6a and 7 (34). This suggests that the VEGFA_189_ isoform is important for endothelial cell migration and that its reduction following enhancer mutation could have an effect on cell proliferation. To demonstrate the physiological relevance of the enhancer-mediated alternative splicing of VEGFA, we monitored the proliferation rate of the parental K562 cells and the enhancer mutated clones 9 and 26. Our results demonstrate that mutations at VEGFA +157 enhancer in clones 9 and 26 resulted in slower proliferation rate (Figure 2C). This suggests that the level of VEGFA expression as well as its alternative splicing is important for cell proliferation in a CML cell line.

### Tethering p300 to the enhancer and promoter

**Figure 3.**
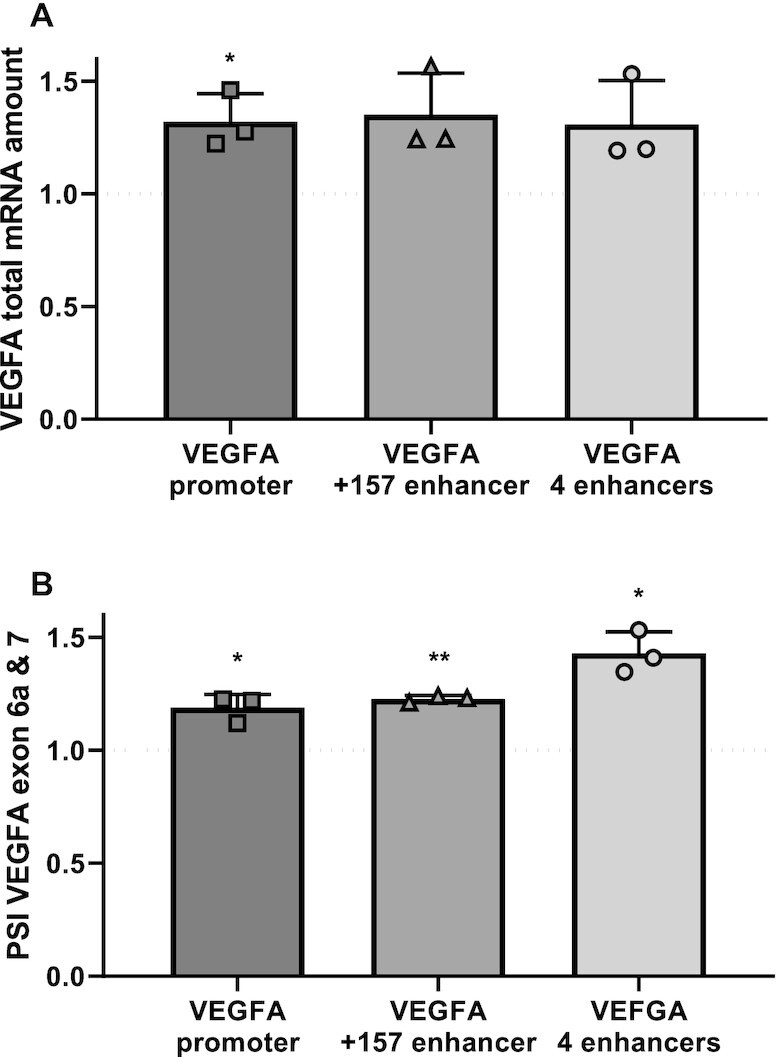
VEGFA +157 enhancer promotes inclusion of VEGFA exons 6a and 7. (**A** and**B**) K562 cells were transfected with either dCas9-p300 core (mut) or dCas9-p300 core (WT) with four gRNAs targeted to the VEGFA promoter or +157 enhancer or with a single gRNA targeting each of four VEGFA enhancers marked in Figure 1B for 30 h. Total RNA was extracted and analyzed by real-time PCR for total mRNA of VEGFA relative to *CycloA* and *hTBP* reference genes (A) and for VEGFA_121_ and VEGFA_189_ relative to VEGFA total mRNA amount. PSI was calculated by VEGFA_189_ relative to VEGFA_121_ (**B**). Values are expressed as dCas9-p300 core (WT) relative to dCas9-p300 core (mut) and horizontal broken lines indicate no change between dCas9-p300 core (WT) relative to dCas9-p300 core (mut). Plots represents the mean of three independent experiments and ± SD (*P< 0.05; **P< 0.01).

To strengthen the link between enhancer activity and alternative splicing, we sought to activate the VEGFA +157 enhancer. We used the nuclease-deficient Cas9 (dCas9) conjugated to the p300 enzymatic core (35). The CRISPR/dCas9 system allows us to tether a chromatin protein core domain to specific chromosome locations using a pool of four gRNAs. A pool of gRNAs was shown empirically to perform better than a single gRNA (35). We tethered p300’s enzymatic core to the +157 validated enhancer as well as additional three putative enhancers predicted by both the H3K27ac and the Hi-C data (Figure 1B, green arrows with yellow dot). In this case we used a single gRNA to each location. We co-transfected K562 cells with dCas9-p300 core (mut) or dCas9-p300 core (WT) and four gRNA plasmids targeting either the VEGFA promoter, the +157 enhancer or the four enhancers. Our results demonstrate that tethering p300’s enzymatic core to either the VEGFA promoter, the +157 enhancer or the four enhancers up-regulates VEGFA total mRNA by 20–30% (Figure 3A). This moderate change in expression may be due to a low dynamic range in K562 cells whose expression of VEGFA is very high and the chromatin at the promoter and enhancer is highly accessible. Accessibility of the chromatin can be demonstrated by acetylation of H3K27 and DHS sites at these regions (Figure 1B). In addition to total mRNA amount, we measured the amount of the enhancer RNA (eRNA) generated by the +157 enhancer. We designed primers to the 3188 nt long eRNA (GH06J043925) (Supplementary Figure S1B) predicted in the +157 region and found similar levels of eRNA and also of VEGFA nascent mRNA (Supplementary Figure S3A and B). Monitoring VEGFA alternative splicing following tethering of p300 to the promoter or enhancer showed inclusion of VEGFA exons 6a and 7 (Figure 3B). These results are in line with our previous results, with the mutated +157 enhancer, suggesting that VEGFA promoter and +157 enhancer promote inclusion of exons 6a and 7 giving rise to VEGFA_189_ and a reduction in VEGFA_121_. We have previously showed that VEGFA_189_ induces endothelial cell migration (34), and thus our results might point to the VEGFA +157 enhancer as promoter of expression and pro-angiogenic isoforms.

### High expression of +157 eRNA and inclusion of exons 6a and 7 in CML patients

**Figure 4.**
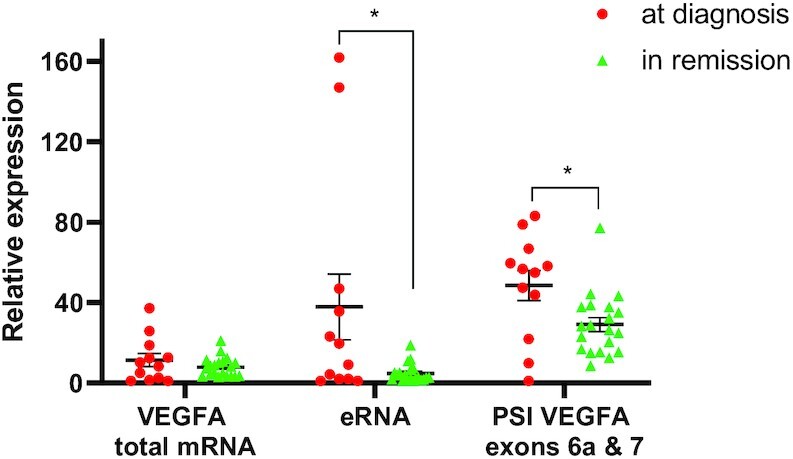
CML patients have greater inclusion of VEGFA exons 6a and 7 at diagnosis. RNA was extracted from peripheral blood samples collected from 12 CML patients at diagnosis (BCR-ABL positive, red circles, 1 sample per patient) and during remission (BCR-ABL negative, green triangles, 1–3 samples per patient). RNA was analyzed by real-time PCR for total mRNA amount of VEGFA and VEGFA +157 eRNA relative to *CycloA* and *hTBP* reference genes and for VEGFA_121_ and VEGFA_189_ relative to VEGFA total mRNA amount. PSI was calculated by VEGFA_189_ relative to VEGFA_121_.

VEGFA protein is secreted from CML cells, which leads to high levels of VEGFA in the plasma of patients (19,36). We asked if overexpression of VEGFA in CML patients is accompanied by a change in alternative splicing. We extracted RNA from peripheral blood of twelve patients at different stages of their disease, represented by BCR-ABL fusion gene quantification (Supplementary Table S1). Although we could not detect significantly higher expression levels of VEGFA total mRNA at diagnosis, we identified significant overexpression of the +157 enhancer eRNA in a subset of patients, suggesting activation of the enhancer (Figure 4). Specifically, our results show that both enhancer activation and inclusion of exons 6a and 7 are reduced when patients are in remission, suggesting that in CML patients, activity of the +157 enhancer goes hand in hand with a change in alternative splicing (Figure 4 and Supplementary Figure S4A–D).

### Slow RNAPII elongation rate promotes VEGFA exclusion of exons 6a and 7

**Figure 5.**
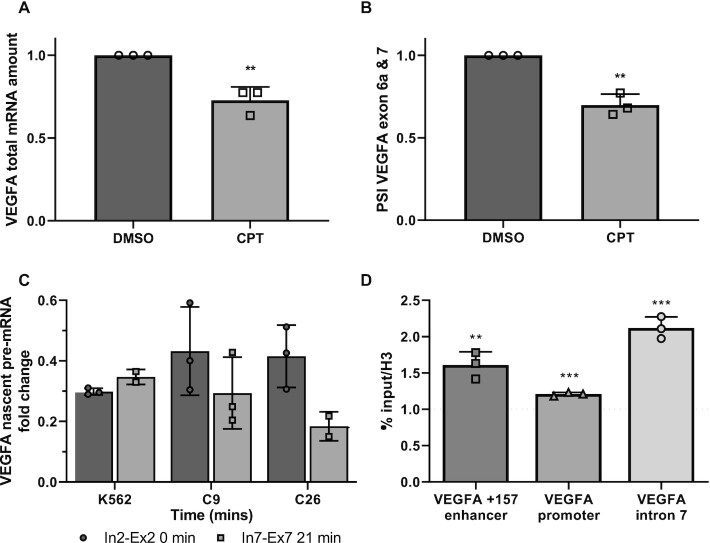
Slow elongation of RNAPII promotes exclusion of VEGFA exons 6a and 7. (**A** and **B**) K562 cells were treated with 6 μM CPT for 6 h. Total RNA was extracted and analyzed by real-time PCR for total mRNA amount of VEGF relative to *CycloA* and *hTBP* reference genes (A) and for VEGFA_121_ and VEGFA_189_ relative to VEGFA total mRNA amount (B). PSI was calculated by VEGFA_189_ relative to VEGFA_121_. (**C**) Nascent mRNA production in WT K562 cells and enhancer mutated C9 and C26 cells in different regions of the *VEGFA* gene after release from DRB-inhibition. (**D**)K562 cells were transfected with either dCas9-p300 core (mut) or dCas9-p300 core (WT) with four gRNAs targeted to the VEGFA promoter or +157 enhancer for 30 h. ChIP was performed of H3 pan-acetyl at the VEGFA enhancer, promoter and intron 7 following tethering to the +157 enhancer. Values represent averages of three independent experiments ± SD and are expressed as dCas9-p300 core (WT) relative to dCas9-p300 core (mut) (**P< 0.01, ***P< 0.001).

Reduced expression of VEGFA as a result of enhancer +157 mutation could be due to less transcription initiation and/or slow RNAPII elongation kinetics along the gene. To examine the role of RNAPII elongation rate in the alternative splicing of VEGFA, we slowed RNAPII using a low concentration of CPT (6 μM) in K562 cells. Our results show that total VEGFA mRNA and eRNA were lower but no change in nascent mRNA was detected (Figure 5A; Supplementary Figure S5A and B). In addition, we found that slowing RNAPII elongation rate promotes exclusion of VEGFA exons 6a and 7 (Figure 5B). Furthermore, we co-transfected HEK293T cells with plasmids expressing α-amanitin-resistant polymerases, a mutant ‘slow’ RNAPII (mutant C4) or WT RNAPII (37). α-Amanitin blocks only endogenous RNAPII transcription as the WT and ‘slow’ plasmids are resistant to it. We assessed the alternative splicing pattern of VEGFA generated by transcription with the recombinant polymerases. Our results show that total VEGFA mRNA and VEGFA nascent RNA were lower with no change in eRNA, and in addition we observed exclusion of VEGFA exons 6a and 7 (Supplementary Figure S5C–F). These findings are similar to our findings with VEGFA +157 mutated enhancer (Figure 2B). This suggests that the *VEGFA* enhancer promotes reduced RNAPII elongation rate to affect both gene expression and alternative splicing of the *VEGFA* gene.

### VEGFA +157 enhancer regulates RNAPII elongation rate

To verify our hypothesis that the *VEGFA* enhancer regulates RNAPII elongation rate, we measured elongation rate in K562 cells and the enhancer mutated clones 9 and 26. To this end we arrested RNAPII at promoter-proximal sites by treating with DRB and measured VEGFA nascent pre-mRNA (intron-exon junction) after the drug was washed out. We compared pre-mRNA levels of exon 7 region with exon 2 region to determine RNAPII elongation rate in the three cell lines. K562 cells incubated with DRB were able to recover transcription of the exon 7 region to that of the levels of exon 2 of the *VEGFA* gene within 21 min of DRB removal while recovery was 28 min or longer in the mutated clones 9 and 26 (Figure 5C and Supplementary Figure S5G–I). These results suggest that VEGFA +157 enhancer regulates RNAPII elongation rate.

RNAPII elongation rate is determined by the level of pause duration at the gene body (38), which is affected by nucleosomes (39,40). Acetylation of the nucleosomes weakens the nucleosome binding to the DNA and was shown to reduce pause duration and thus increase elongation rate. To strengthen our hypothesis that the +157 enhancer regulates RNAPII elongation rate, we measured histone 3 acetylation in VEGFA’s gene body following tethering of p300’s enzymatic core to the +157 enhancer. To this end we co-transfected K562 cells with dCas9-p300 core (mut) or dCas9-p300 core (WT) and four gRNA plasmids targeting the +157 enhancer and performed ChIP-qPCR using H3 and H3 pan acetyl (H3ac) antibody. Our results showed an increase of 50% in H3ac at the tethered region and a much smaller increase at the *VEGFA* promoter region (Figure 5D). Interestingly we measured twice as much H3ac at the gene body specifically at intron 7 (Figure 5D). This result suggests that the +157 enhancer regulates RNAPII elongation rate by acetylating the gene body to allow for reduced RNAPII pausing. In addition, histone acetylation at the vicinity of the alternatively spliced exons can also take part in VEGFA alternative splicing regulation mediated by the enhancer (41,42).

### Chromatin factors role in VEGFA alternative splicing

**Figure 6.**
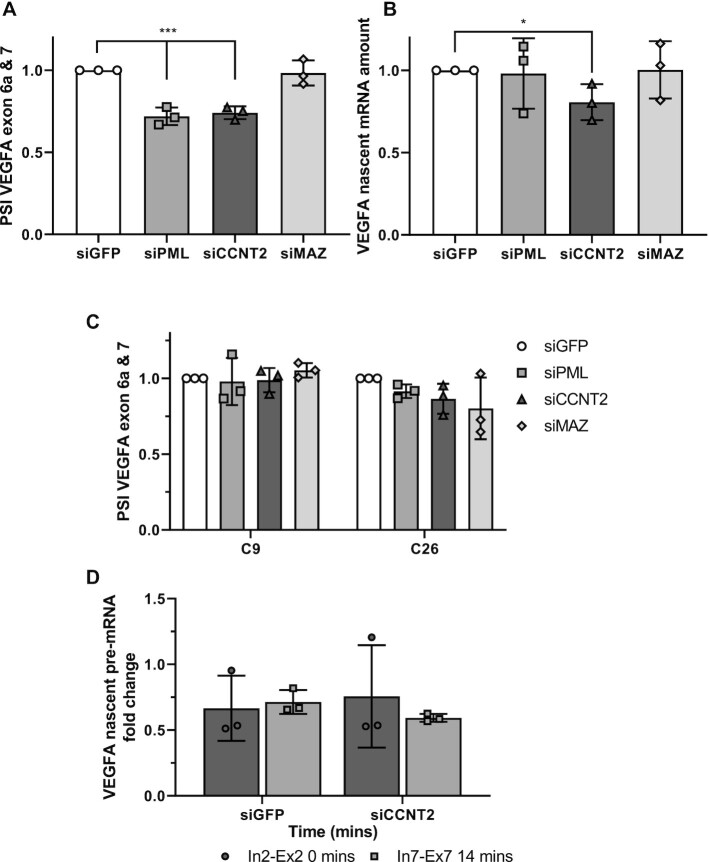
CCNT2 binds VEGFA promoter and enhancer to regulate *VEGFA* elongation rate and alternative splicing. (**A** and**B**) K562 cells were transfected with siRNA against GFP as negative control and siRNA targeting PML, CCNT2 and MAZ for 72 h. RNA was analyzed by real time PCR for VEGFA_121_ and VEGFA_189_ relative to VEGFA total mRNA amount. PSI was calculated by VEGFA_189_ relative to VEGFA_121_ (A); and for nascent pre-mRNA VEGFA relative to VEGFA total mRNA amount (B). (**C**) Enhancer mutated clones 9 and 26 were transfected with siRNA against GFP as negative control and siRNA targeting PML, CCNT2 and MAZ for 72 h. RNA was analyzed by real time PCR for VEGFA_121_ and VEGFA_189_ relative to VEGFA total mRNA amount. PSI was calculated by VEGFA_189_ relative to VEGFA_121_. (**D**) Nascent mRNA production in WT K562 cells transfected with siRNA against GFP as negative control and siRNA targeting CCNT2 in different regions of the *VEGFA* gene after release from DRB-inhibition. Values represent mean ± SD of three independent experiments (* *P*< 0.05; ***P< 0.001).

Transcription factors are present in the spliceosome and are predicted to have a role in alternative splicing (43,44). To search for factors mediating the enhancer's effect on alternative splicing, we analyzed 161 ChIP-seq experiments for DNA binding factors in K562 cells (45). We set the cutoff of the signal binding normalized scores (in the range of 0–1000) to 350 and identified promyelocytic leukemia (PML), cyclin T2 (CCNT2) and MYC-associated zinc finger protein (MAZ) as binding to both the +157 enhancer and the promoter of the VEGFA gene (Supplementary Table S2; Supplementary Figure S6A and B). PML co-localizes to PML nuclear bodies where it functions as a tumor suppressor (46). PML has multiple binding partners with roles in transcription regulation as well as cell cycle arrest, apoptosis, senescence, DNA repair and intermediary metabolism (46). CCNT2 is a cyclin that acts with its kinase partner CDK9 to regulate RNAPII transcription elongation (47,48). MAZ is a transcription factor with a known DNA binding motif and targets (49). To check whether those proteins have a role in VEGFA alternative splicing, we silenced each of the factors in K562 cells with a pool of four siRNA oligos (Supplementary Figure S6C–F). Our results show that no significant change was detected in VEGFA total mRNA amount but that PML and MAZ silencing caused a mild reduction in eRNA levels (Supplementary Figure S6G and H). In addition, we detected exclusion of VEGFA exons 6a and 7 when silencing PML and CCNT2 but not when silencing MAZ (Figure 6A). In addition, CCNT2 is a positive regulator of RNAPII elongation kinetics (47,48), and its silencing did yield a mild reduction in VEGFA nascent mRNA (Figure 6B). Our results point to PML and CCNT2 as regulators of VEGFA alternative splicing, and their effect of exclusion of VEGFA exons 6a and 7, when silenced, is similar to that of mutating the +157 VEGFA enhancer (Figure 2B).

### CCNT2 is a regulator of RNAPII elongation and alternative splicing of *VEGFA*

**Figure 7.**
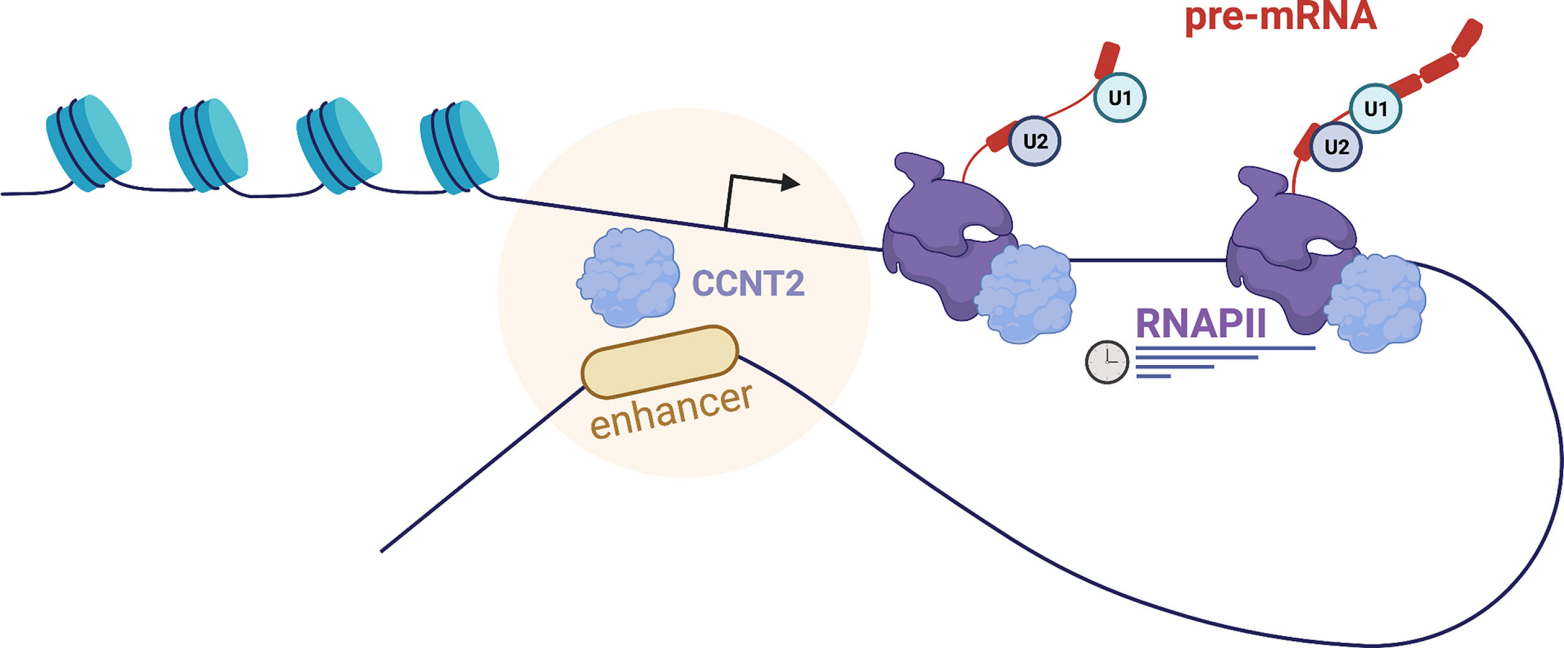
Schematic representation of the proposed mechanism. The RNAPII elongation facilitator CCNT2 binds to *VEGFA* promoter and enhancer and drives inclusion of exons b6 and 7. Spliceosome assembly on the nascent pre-mRNA is depicted by U1 and U2 short non-coding RNA.

The similar effect of siPML and siCCNT2 silencing and mutating of the +157 VEGFA enhancer on alternative splicing could suggest that the mutations in clones 9 and 26 are in close proximity to PML and CCNT2 binding sites. Indeed, the mutation and the protein binding sites are found within the same DHS region (150 nt long) in close proximity (Supplementary Figure S6I). To check the effect of the mutations in clones 9 and 26 on PML and CCNT2 activity, we silenced each of these factors in C9 and C26 cells. We found no significant change in alternative splicing of VEGFA in the mutant cell lines, which suggests that the sequence mutated is important for PML and CCNT2 function in the +157 enhancer (Figure 6C and Supplementary Figure S6J–L). To check whether CCNT2 regulates *VEGFA* elongation rate in K562 cells, we silenced CCNT2 and measured elongation rate. Our results demonstrate slower elongation rate in cells silenced for CCNT2 as demonstrated by the elongation time in the 10 kb between exon 2 to exon 7 (Supplementary Figure S6M–N). To learn about the connection between RNAPII elongation rate and CCNT2’s role in alternative splicing, we slowed RNAPII elongation rate using a low concentration of CPT (6 μM) in K562 cells with or without silencing of CCNT2. Our results show the same magnitude of alternative splicing change with each of the perturbations both separately and together (Supplementary Figure S6O–R). This result could indicate that silencing of CCNT2 leads to exclusion of VEGFA exons 6a and 7 likely by slowing down RNAPII elongation rate (Figure 7). Thus, our results suggest that the VEGFA enhancer regulates VEGFA splicing by regulating RNAPII elongation rate.

## DISCUSSION

Pre-mRNA splicing occurs co-transcriptionally (50), with the processes being coupled via kinetic and physical interaction of the RNA splicing machinery with RNAPII (44,51). Here we show that manipulating the VEGFA enhancer leads to regulation of its alternative splicing. We identify PML and CCNT2 as possible players mediating the connection of the enhancer’s activity with alternative splicing regulation. CCNT2 releases pausing of RNAPII (47,48) and indeed silencing it in K562 cells led to a mild reduction in VEGFA nascent RNA and slow elongation rate of the *VEGFA* gene in K562 (Figure 6B and D). Our results suggest that binding of CCNT2 to both VEGFA enhancer and promoter allows for fast elongation rate that promotes high gene expression and inclusion of exons 6a and 7 (Figure 6A, B and D and Supplementary Figure S6A and B). Our results suggest that CCNT2 and PML are needed for VEGFA overexpression and thus might be upregulated in CML. While there are no data on CCNT2 in CML, it was shown to be overexpressed in acute myeloid leukemia and to promote proliferation in this cancer (52). PML, while downregulated in many types of cancer, was found to be upregulated in CML and to be a positive regulator of self-renewal in CML-initiating cells (53). Our work adds a level of complexity to alternative splicing regulation and deepens the interplay between transcription and alternative splicing.

VEGFA enhancer is demethylated in various hematopoietic cancers, resulting in higher gene expression (2). Our work suggests that enhancer activation alters VEGFA alternative splicing to produce isoforms that promote angiogenesis. The idea of enhancers making a connection between gene expression and isoform abundance seems highly intuitive on the physiological level. This connection is strengthened by our observation that in CML patients we observed inclusion of exons 6a and 7 (Figure 4), which promotes VEGFA_189_ that we have previously shown to induce endothelial cell migration (34). Finally, VEGFA +157 enhancer promotes VEGFA isoforms that have been shown to promote endothelial cell migration and thus cancer.

Our previous work, as well as the work of others, connected promoter activity to alternative splicing by the acetyltransferase p300 (54,55). Binding of p300 at the promoter region acetylates not only histones at this region, but also splicing factors, so regulating alternative splicing of the specific gene (55). Acetylation of splicing factors can either weaken or strengthen the splicing factors' RNA-binding properties that in turn regulate alternative splicing. Our results tethering p300 to the promoter and +157 enhancer of VEGFA in K562 cells show inclusion of exons 6a and 7 (Figure 3B). This result attests to the activation of the enhancer and its effect on splicing also suggests that acetylating the histones at the promoter and enhancer regions allows stronger binding of transcription factors that then regulate alternative splicing, as we show for PML and CCNT2. Furthermore, this result could also indicate that p300 binding to the enhancer loops it to the promoter to allow for acetylation of splicing factors. Additional work is needed to connect these multiple layers of alternative splicing regulation.

Our work here focuses on one enhancer of the VEGFA gene, but chromatin marks and Hi-C data collected in K562 cells suggest that VEGFA has at least five more enhancers located in proximity to the +157 enhancer (Figure 1B). Hence, the expression of *VEGFA* is the sum of inputs from its multiple enhancers. This could explain the mild change in expression we observed when tethering p300 to the one or even four enhancers of *VEGFA* (Figure 3A). Our hypothesis is that alternative splicing regulation by the enhancer is the sum of several enhancers.

Our results show weak changes in eRNA while manipulating the enhancer. Activating the enhancer by tethering p300 did not show any change in eRNA expression (Supplementary Figure S3A), but silencing PML transcription factor which binds VEGFA promoter and +157 enhancer show a mild reduction in eRNA and VEGFA exclusion of exons 6a and 7 (Figure 6A and B; Supplementary Figure S6H). These results do not form a strong association between the eRNA and alternative splicing and additional experiments are needed to further study this connection.

## DATA AVAILABILITY

The data supporting the findings of this study are available within the article and its supplementary material. Data analyzed in this study is available by ENCODE (32,45).

## Supplementary Material

zcab029_Supplemental_File
