# Supplementary material for: VEGFA’s distal enhancer regulates its alternative splicing in CML
Source: NAR Cancer. 2021 Jul 13;3(3):zcab029. doi: 10.1093/narcan/zcab029 (PMC8276762; doi:10.1093/narcan/zcab029)
Supplement: zcab029_Supplemental_File [file zcab029_supplemental_file.pdf]

A Sup. Figure S1

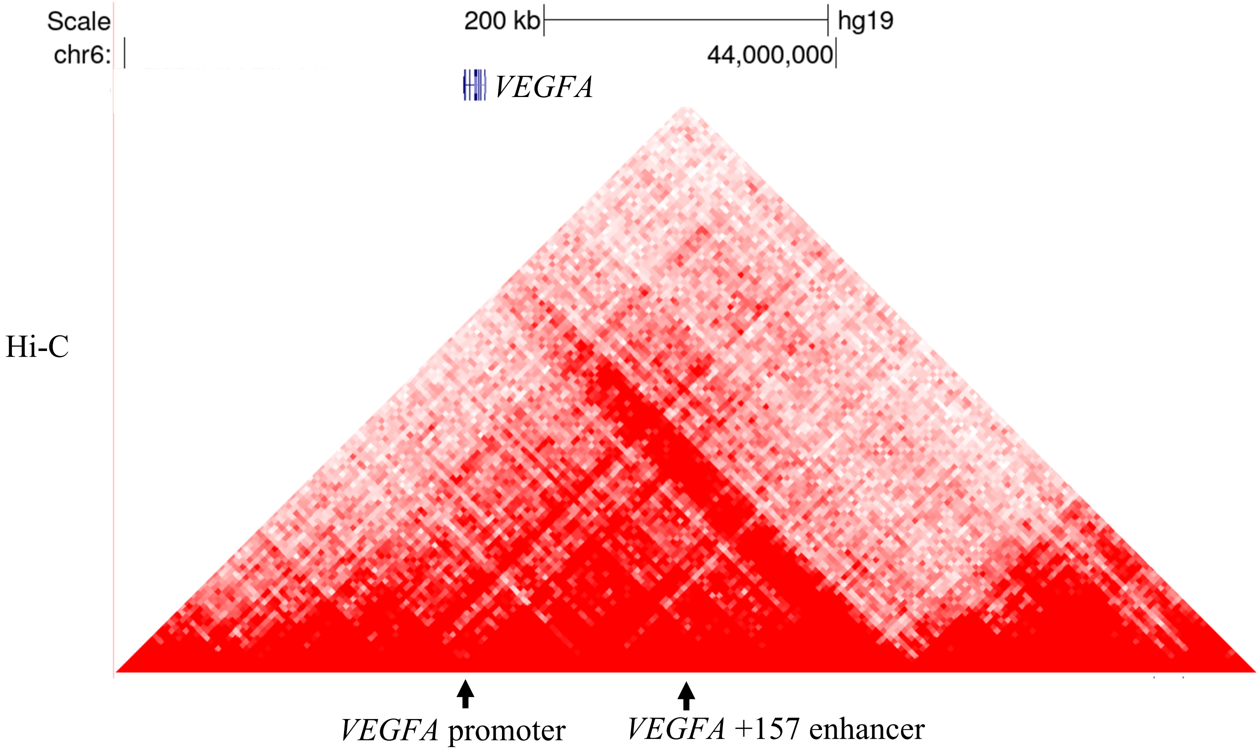

B

K562 WT: CCCTCCTCCCTCTGCCGCCTGAGTCAGAGAAGCCATGTGTTCAAAATCA

Clone 9: CCCTCCTCCCTCTGCCGCCTGAGTCAGAGAA**A**GCCG

Clone 26: CCCTCCTCCCTCTGCCGCCTGA - - - - - - - -   - - - - - - - - - - - - - GTTCAAAATCA
Clone 26: CCCTCCTCCCTCTGCCGCC - - - - - - - - - - -   - - - - -**GGG**ATGTGTTCAAAATCA
Clone 26: CCCTCCTCCCTCTGCCGCCTGAGTCAGAG**A**AAGCC**GGG**ATGTGTTCAAAATCA

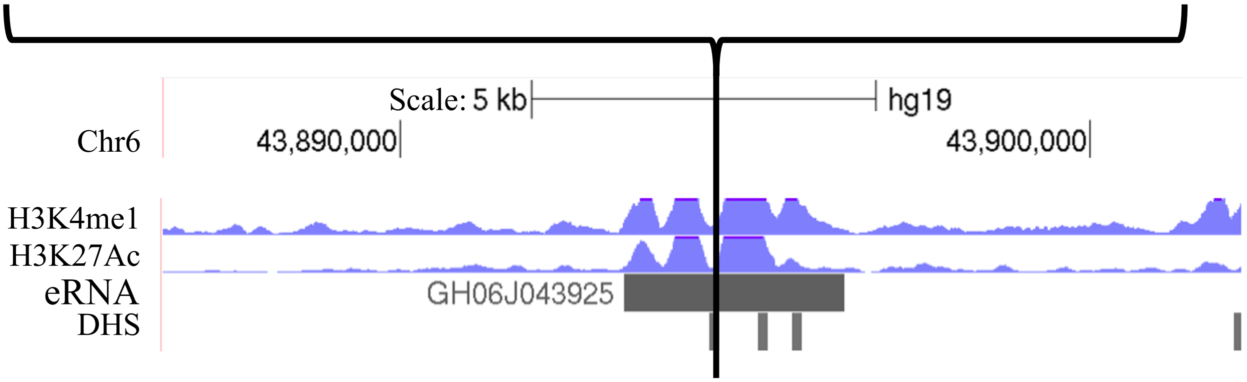

Fig. S1: **A**. ChIP-seq tracks for H3K27ac and H3K4me1 including DHS at the VEGFA locus in K562 cells. VEGFA +157 enhancer is marked by the eRNA (GH06J043925) and is enriched for H3K27ac and H3K4me1. At the bottom part: Hi-C data (5 kb resolution) at the VEGFA locus in K562 cells. Black arrows indicate the *VEGFA* +157 enhancer and promoter. The Hi-C data suggest that the promoter and enhancer are in the same TAD. **B**. Sequence alignments of the VEGF +157 enhancer site showing K562 WT, C9 and C26 clones. black line indicates the location of the mutations at the the VEGFA locus.

Sup. Figure S2:

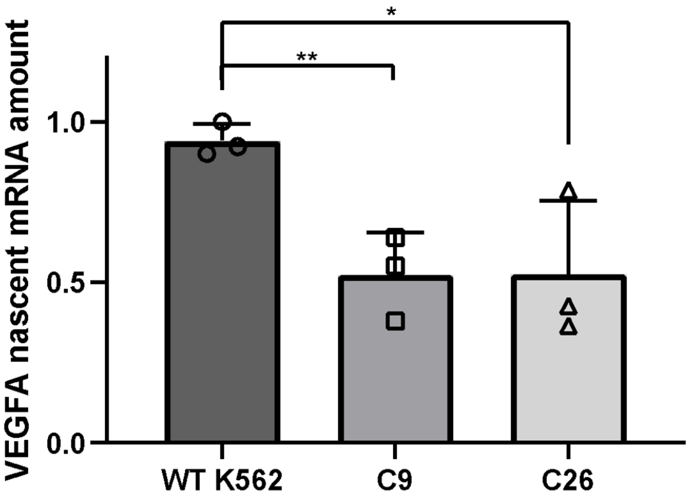

Fig. S2: RNA was extracted from WT K562 cells and enhancer mutated C9 and C26 cells, and analyzed by real-time PCR for nascent pre-mRNA VEGFA relative to VEGFA total mRNA amount.

Sup. Figure S3:

A

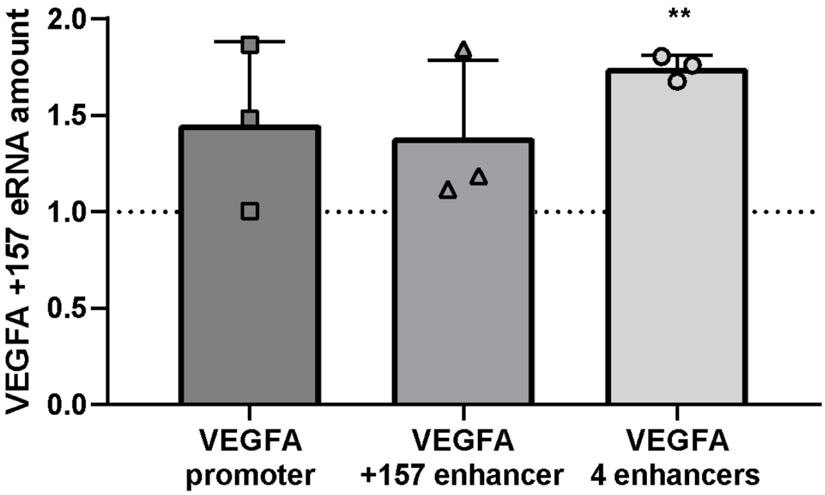

B

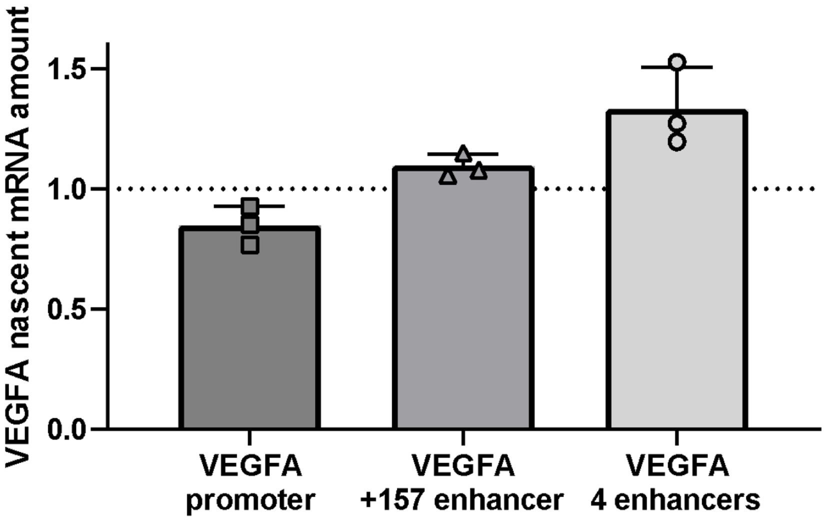

Fig. S3: **A-B.** K562 cells were transfected with either dCas9-p300 core (mut) or dCas9-p300 core (WT) with four gRNAs targeted to the VEGFA promoter or +157 enhancer or with a single gRNA targeting each of four VEGFA enhancers, marked in Fig. 1B, for 30 h. Total RNA was extracted and and analyzed by real-time PCR for total mRNA amount of for VEGFA +157 eRNA relative to *CycloA* and *hTBP* reference genes (**A**); and for nascent pre-mRNA VEGFA relative to VEGFA total mRNA amount (**B**); and horizontal broken lines indicate no change between dCas9-p300 core (WT) relative to dCas9-p300 core (mut). Values are expressed as dCas9-p300 core (WT) relative to dCas9-p300 core (mut). Plots represents the mean of three independent experiments and ±SD (* p<0.05; ** p<0.01).

Sup. Figure S4:

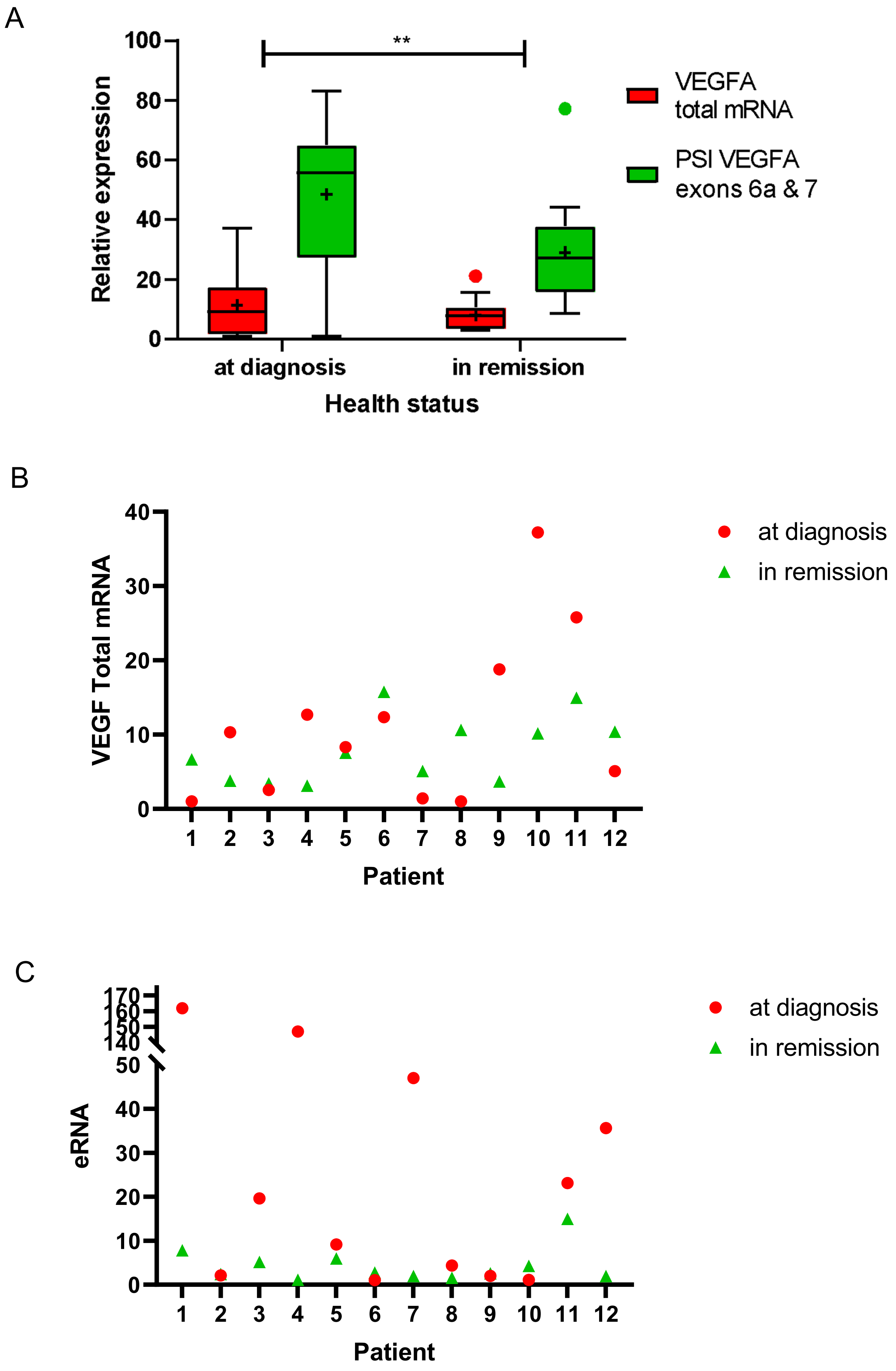

D

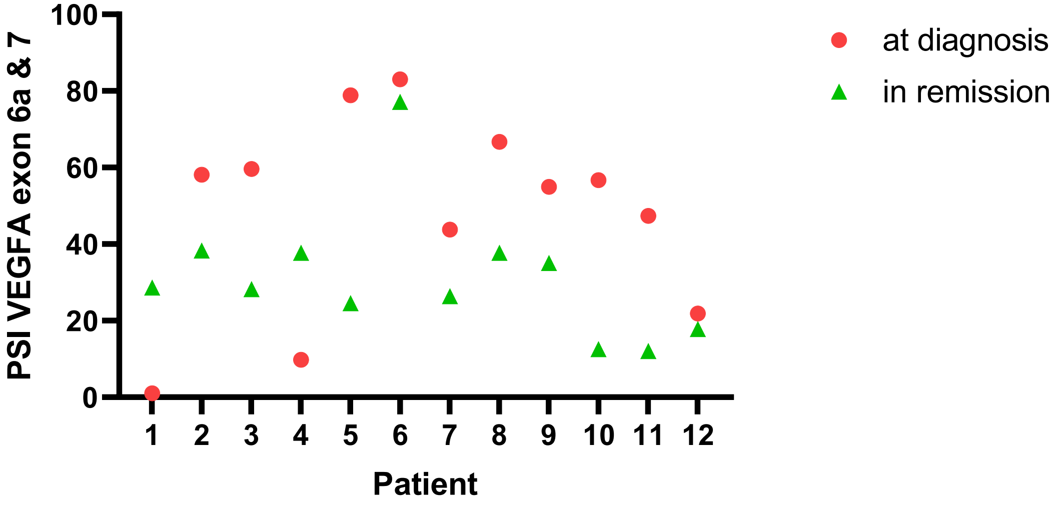

Fig. S3: RNA was extracted from peripheral blood samples collected from 12 CML patients at diagnosis (BCR-ABL positive, diagnosis, 1 sample per patient) and during remission (BCR-ABL negative, remission, 1-3 samples per patient). RNA was analyzed by real time PCR for total mRNA amount of VEGFA relative to *CycloA* reference gene, VEGFA +157 eRNA relative to *CycloA* and *hTBP* reference genes and for $VEGFA_{121}$ and $VEGFA_{189}$ relative to VEGFA total mRNA amount. PSI was calculated by $VEGFA_{189}$ relative to $VEGFA_{121}$. **A**. The results are represented as a Tukey boxplot. Analysis by mixed effects model (REML) showed a significant difference in relative expression between diagnosis and remission (predicted mean at diagnosis: 29.78; predicted mean in remission: 18.50; difference between predicted means: 11.28 ± 3.78, 95% CI of difference 3.45-19.12, p=0.0068). **B-D**. The results are shown for each individual patient at diagnosis (red dot; single measurement) and in remission (green triangle; average of 1-3 measurements) for total VEGFA mRNA (**B**), VEGFA +157 eRNA (**C**) and VEGFA exon 6a & 7 inclusion (**D**). Paired t tests were performed on each set of data (comparing the values for each patient at diagnosis to that in remission). A significant difference was only found for VEGFA exon 6a & 7 inclusion.

Sup. Figure S5:

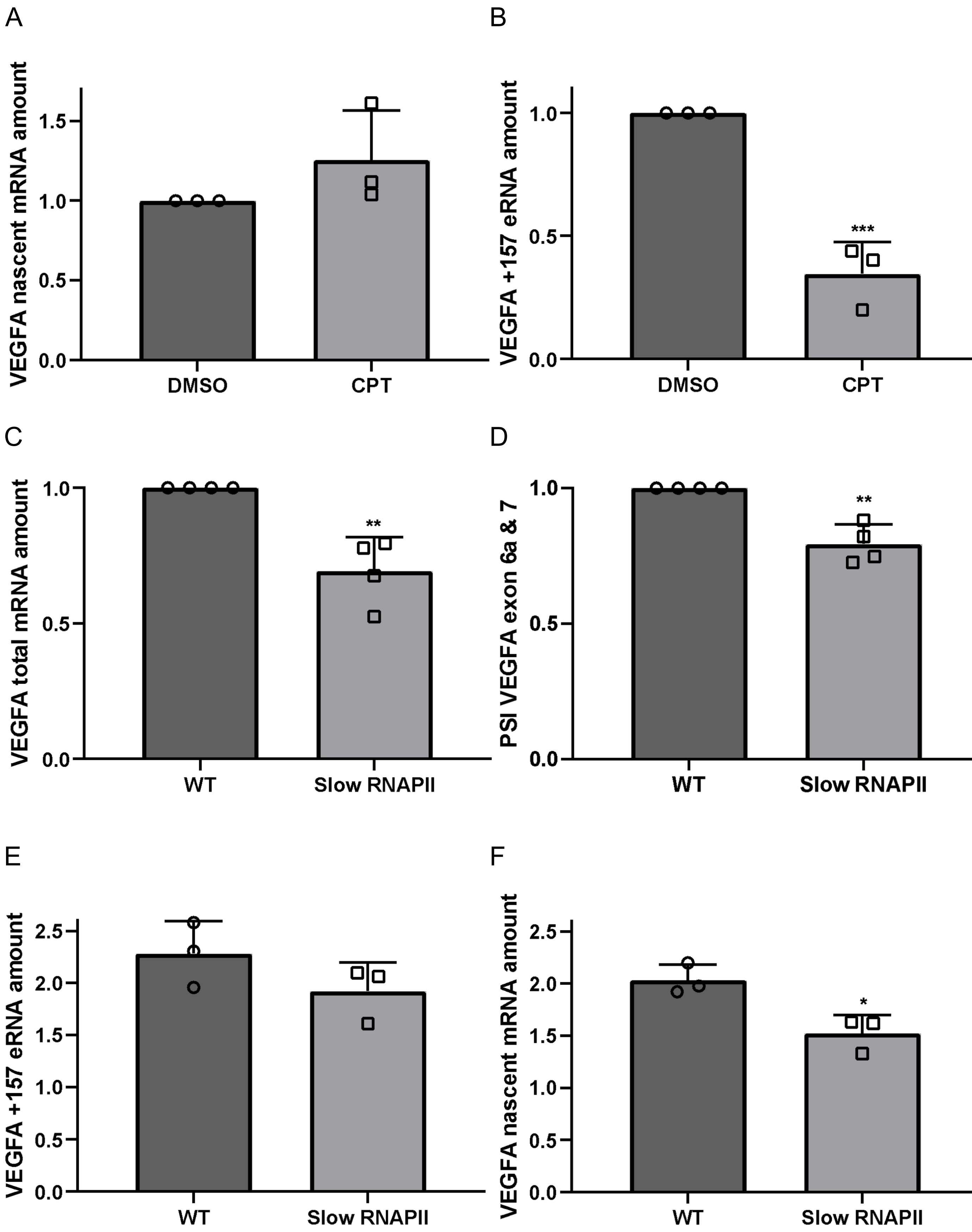

Continue, Sup. Figure S5:

G

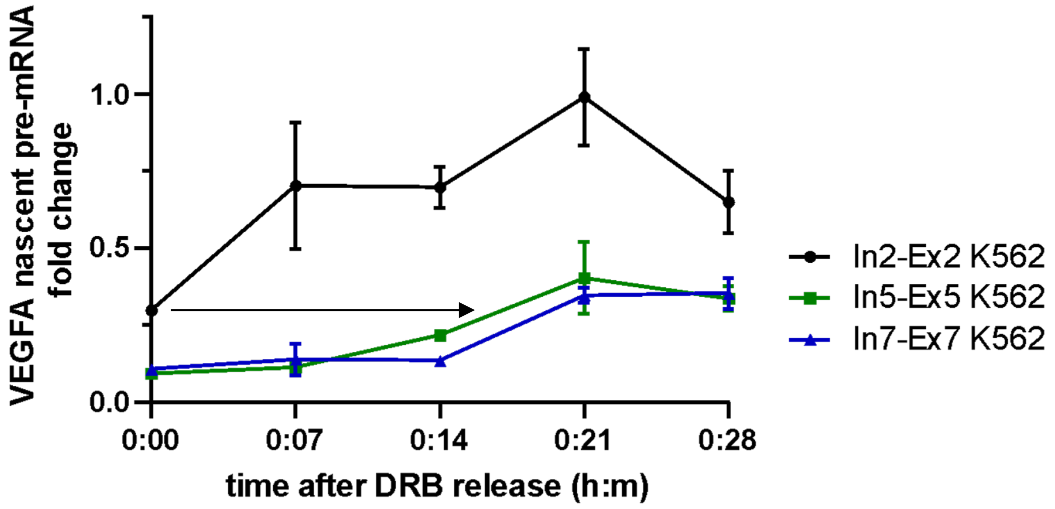

H

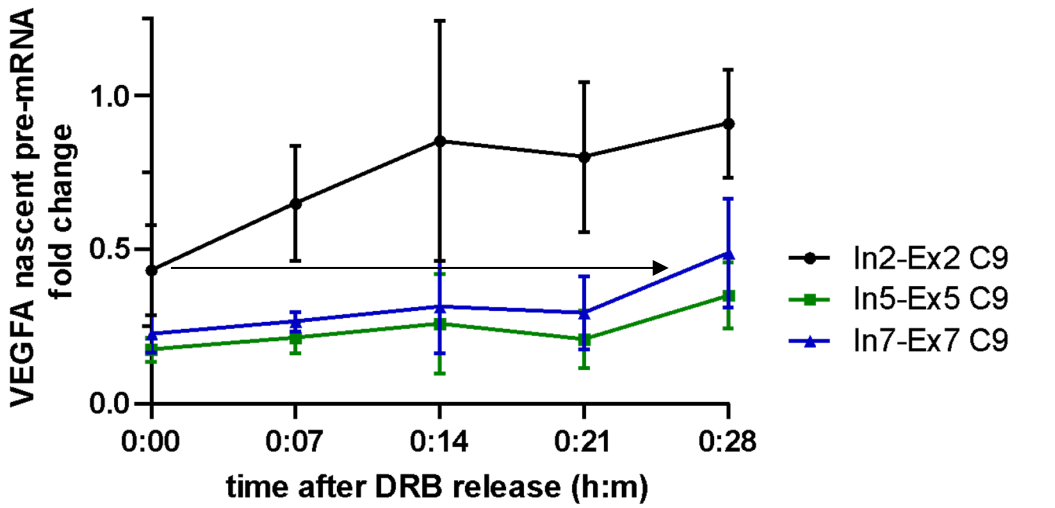

I

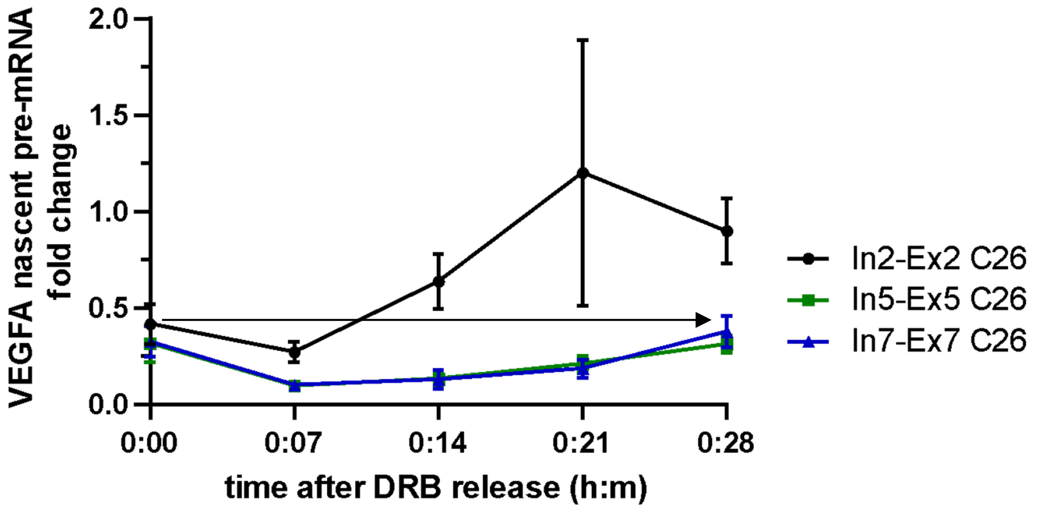

Fig. S5: **A-B.** K562 cells were treated with 6 μM CPT for 6 h. Total RNA was extracted and analyzed by real-time PCR for for nascent pre-mRNA VEGFA relative to VEGFA total mRNA amount (**A**) and for VEGFA +157 eRNA relative to *CycloA* and *hTBP* reference genes (**B**). **C-F**. HEK293T cells were transfected with either WT RNAPII or slow RNAPII and after 24 h treated with α-amanitin for 24 h. Total RNA was extracted and analyzed by real-time PCR for total mRNA amount of VEGF relative to *CycloA* and *hTBP* reference genes (**C**) and for $VEGFA_{121}$ and $VEGFA_{189}$ relative to VEGFA total mRNA amount. PSI was calculated by $VEGFA_{189}$ relative to $VEGFA_{121}$ (**D**) and for VEGFA +157 eRNA relative to *CycloA* and *hTBP* reference genes (**E**) and for nascent pre-mRNA VEGFA relative to VEGFA total mRNA amount (**F**). **G-I.** Nascent mRNA production in WT K562 cells (**G**) and enhancer mutated C9 (**H**) and C26 cells (**I**) in different regions of the *VEGFA* gene after release from DRB-inhibition. Arrow marks the elongation time at the 10 kb spanning from *VEGFA* In2-Ex2 to In7-Ex7.

Sup. Figure S6:

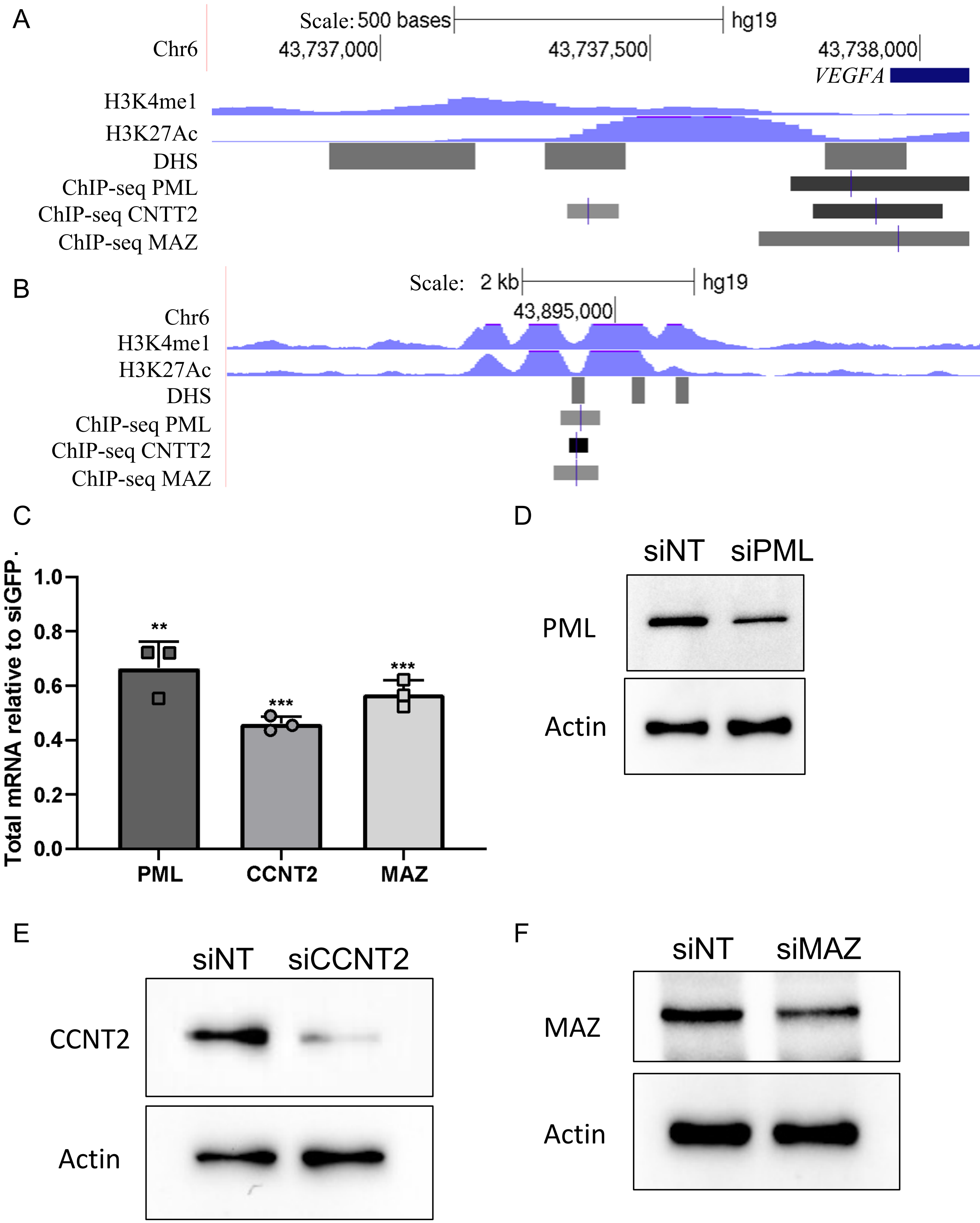

Continue Sup. Figure S6:

G.

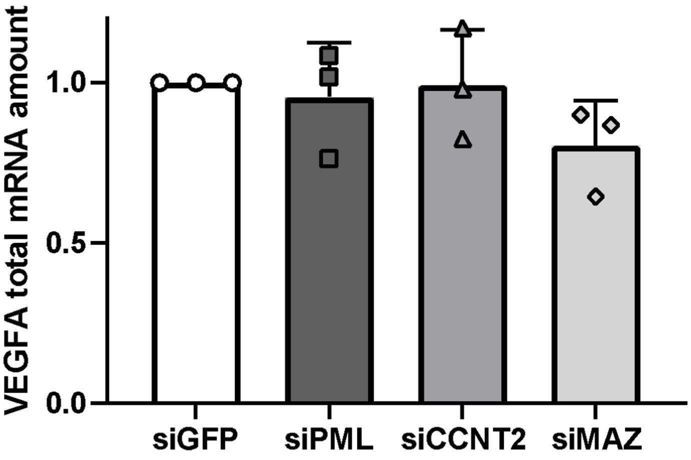

H.

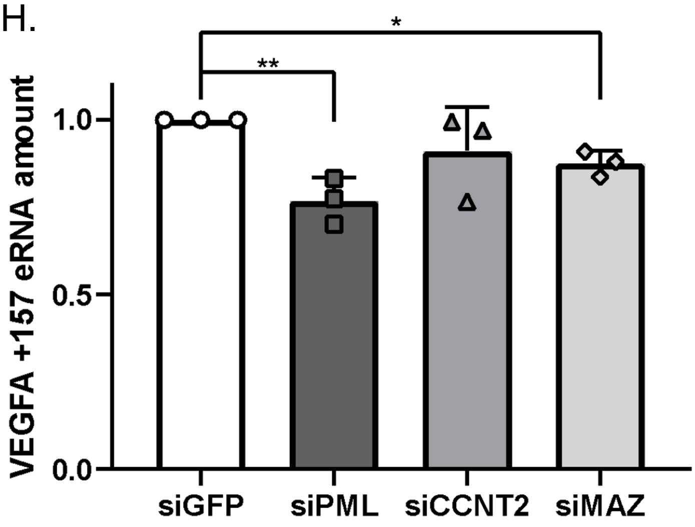

Continue, Sup. Figure S6:

I

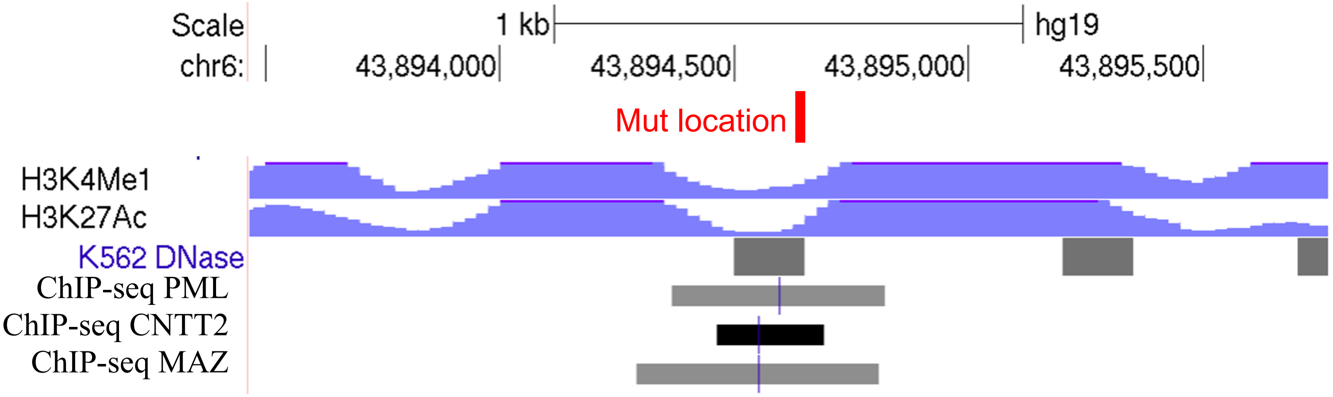

J

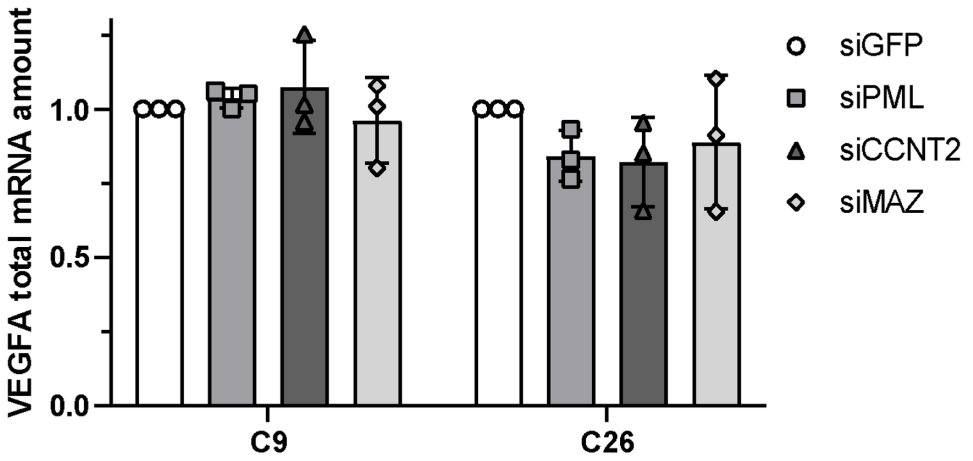

K

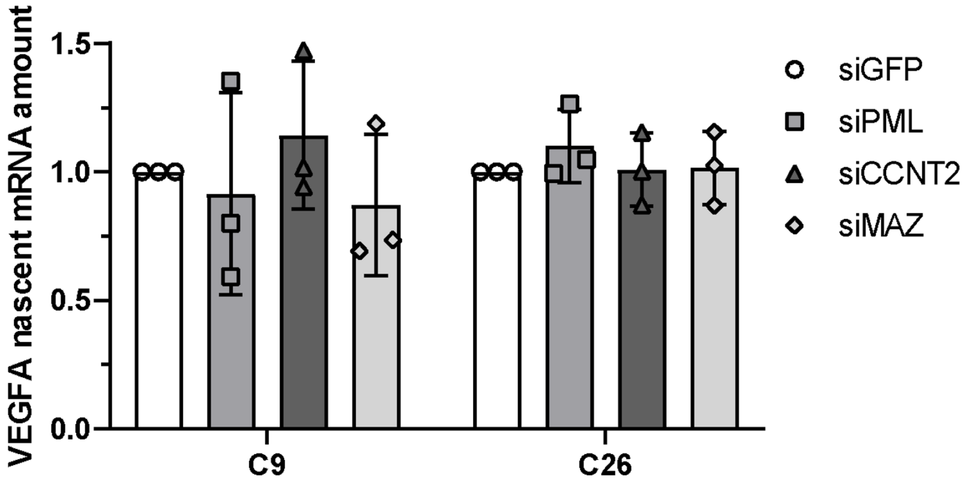

Continue, Sup. Figure S6:

L

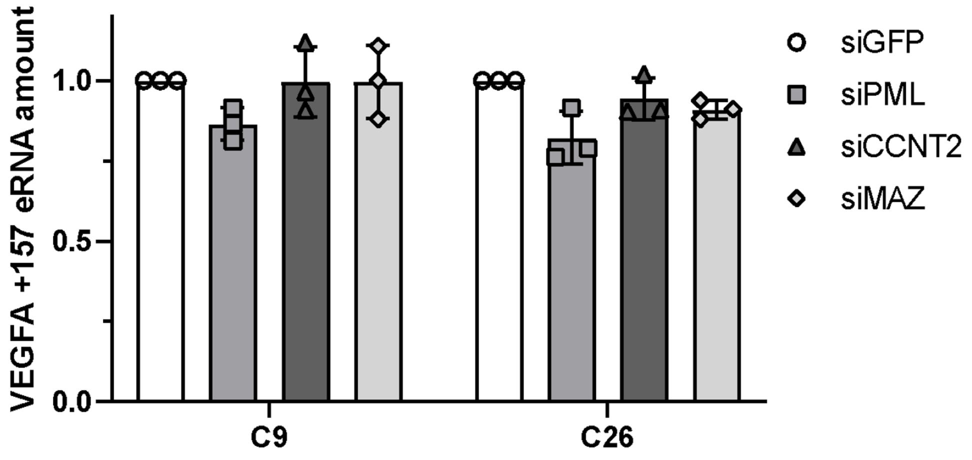

M

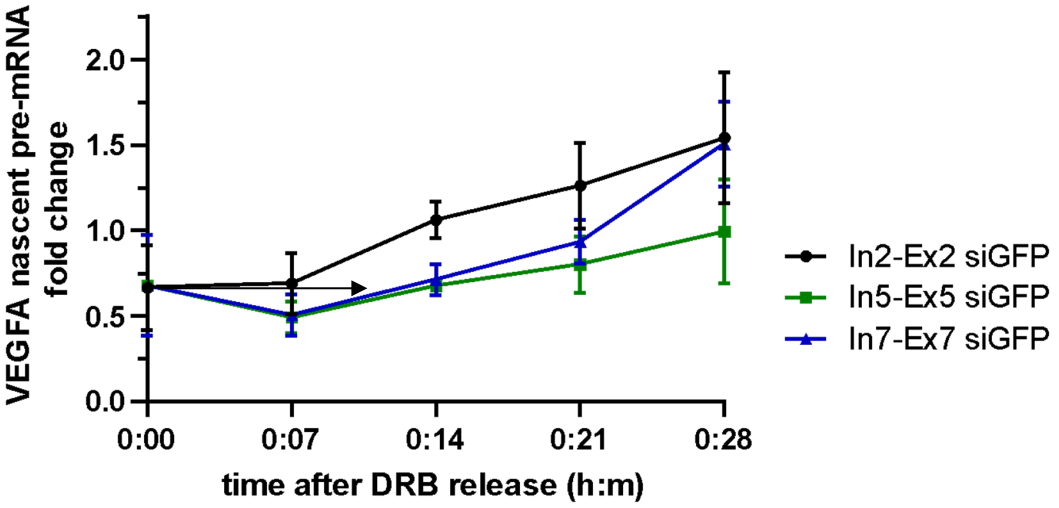

N

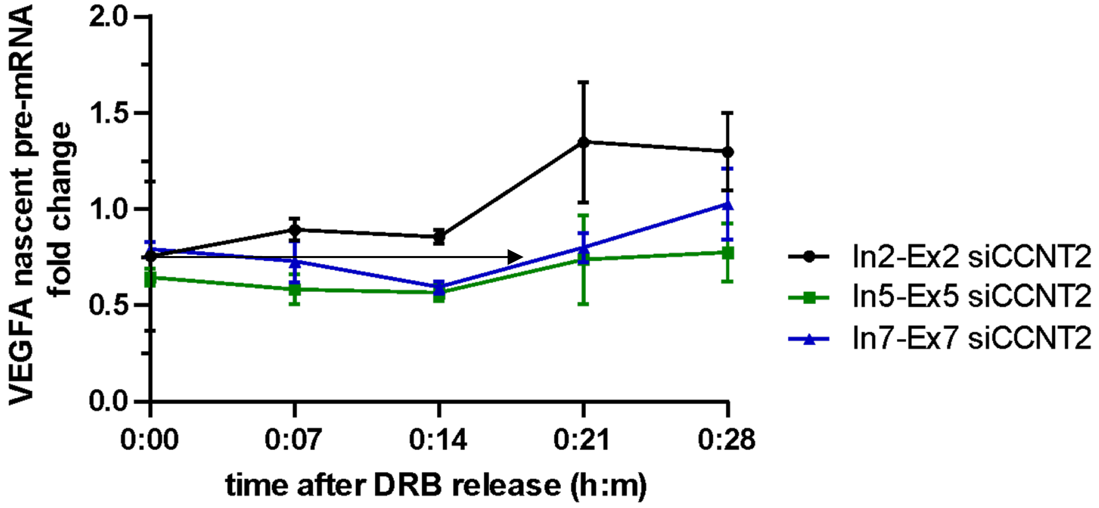

O

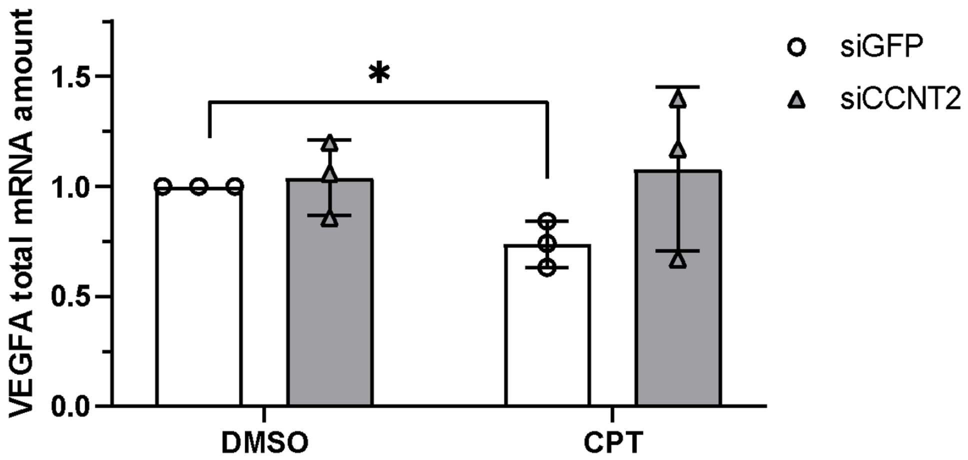
VEGFA total mRNA amount
0.0
0.5
1.0
1.5
*
DMSO
CPT
siGFP
siCCNT2

P

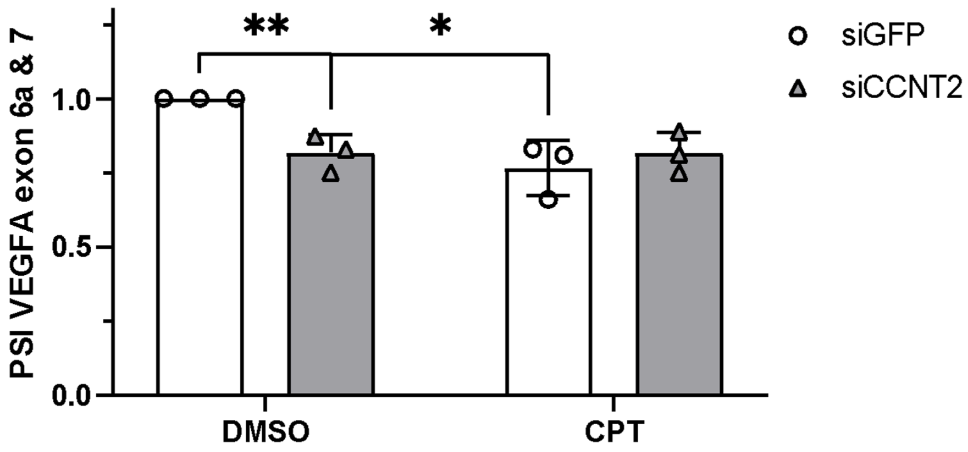
PSI VEGFA exon 6a & 7
0.0
0.5
1.0
**
*
DMSO
CPT
siGFP
siCCNT2

Q

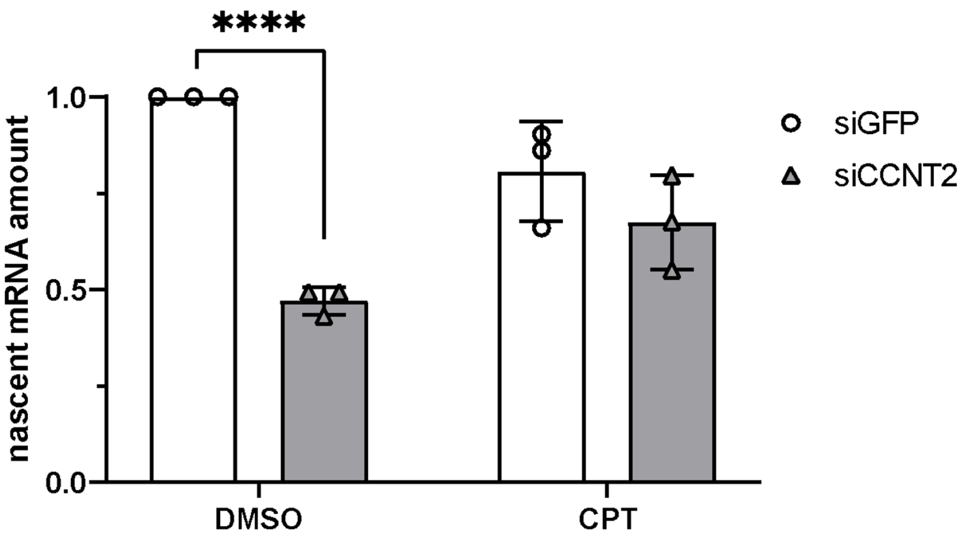
nascent mRNA amount
0.0
0.5
1.0
****
DMSO
CPT
siGFP
siCCNT2

R

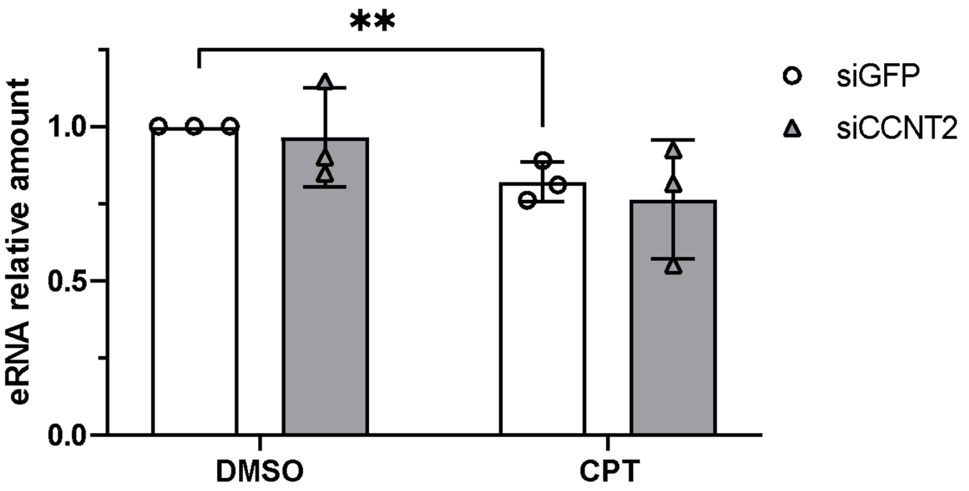

Fig. S6: **A-B**. ChIP-seq tracks for H3K27ac, H3K4me1, PML, CNTT2 and MAZ including DHS and at the VEGFA promoter (**A**) and VEGFA +157 enhancer (**B**) locus in K562 cells. **C-H**. K562 cells were transfected with siRNA against GFP as negative control and siRNA targeting PML, CNTT2 and MAZ for 72 h. RNA was analyzed by real time PCR for PML, CNTT2 and MAZ (**C**) and immunoblotting was conducting using the indicated antibodies (**D-F**) and for VEGFA total mRNA amount relative to *CycloA* and *hTBP* reference genes (**G**) and for VEGFA +157 eRNA relative to *CycloA* and *hTBP* reference genes (**H**). **I.** ChIP-seq tracks for H3K27ac, H3K4me1, PML, CNTT2 and MAZ including DHS and at the *VEGFA* +157 enhancer locus in K562 cells. The location of the mutations in C9 and C26 is marked in red. **J-L**. C9 and C26 cells were transfected with siRNA against GFP as negative control and siRNA targeting PML, CNTT2 and MAZ for 72 h. RNA was analyzed by real time PCR for VEGFA total mRNA amount relative to *CycloA* and *hTBP* reference genes (**J**) and for $VEGFA_{121}$ and $VEGFA_{189}$ relative to VEGFA total mRNA amount, and for nascent pre-mRNA VEGFA relative to VEGFA total mRNA amount (**K**) and for VEGFA +157 eRNA relative to *CycloA* and *hTBP* reference genes (**L**). **M&N.** Nascent mRNA production in WT K562 cells transfected with siRNA against GFP as negative control (**M**) and siRNA targeting CNTT2 (**N**) in different regions of the VEGFA gene after release from DRB-inhibition. Arrow marks the elongation time at the 10 kb spanning from *VEGFA* In2-Ex2 to In7-Ex7. **O-R**. K562 cells were treated with 6 μM CPT for 6 h and transfected with siRNA against GFP as negative control and siRNA targeting CNTT2. Total RNA was extracted and analyzed by real-time PCR for total mRNA amount of VEGF relative to *CycloA* and *hTBP* reference genes (**O**) and for $VEGFA_{121}$ and $VEGFA_{189}$ relative to VEGFA total mRNA amount. PSI was calculated by $VEGFA_{189}$ relative to $VEGFA_{121}$ (**P**) and for VEGFA +157 eRNA relative to *CycloA* and *hTBP* reference genes (**Q**) and for nascent pre-mRNA VEGFA relative to VEGFA total mRNA amount (**R**). Values represent mean ± SD of three independent experiments (* $p<0.05$; ** $p<0.01$, *** $p<0.001$).

Supplementary Table S1: Details of patients and BCR-ABL %

patient #	gender	age	# of samples	BCR-ABL %
1	male	42	4	43%
				neg
				neg
				neg
2	female	42	3	43%
				neg
				neg
3	female	44	2	66%
				1.10%
4	female	40	3	100%
				neg
				0.01%
5	female	31	3	31%
				0.01%
				0.01%
6	male	33	2	40%
				0.5%
7	male	56	3	77%
				neg
				0.007%
8	female	84	2	53%
				0.1%
9	female	16	2	52%
				0.02%
10	male	86	2	116%
				0.008%
11	female	55	3	78%
				0.001%
				0.002%
12	female	56	4	100%
				neg
				neg
				neg

Supplementary Table S2: List of transcription and chromatin factors binding to *VEGFA* promoter and +157 enhancer		
Transcription factor name	normalized scores (in the range 0-1000) in *VEGFA* +157 enhancer (hg19:chr6:43893266-43896453)	normalized scores (in the range 0-1000) in *VEGFA* promoter (hg19:chr6:43,737,336-43,737,845)
CCNT2	446	736
MAZ	1000	390
PML	408	503
ARID3A	789	0
ATF1	415	0
ATF3	883	0
BHLHE40	647	0
CEBPB	1000	0
CHD1	0	0
CHD2	0	0
CTCF	147	1000
CTCFL	0	236
E2F4	0	125
E2F6	154-307	195-517
EGR1	349	657
ETS1	0	242
FOS	142-280	0
FOSL1	189	0
GATA1	395	0
GATA2	281	0
GTF2B	109	0
GTF2F1	0	166
HDAC1	189	0
HDAC2	142	0
HDAC8	201	0
HMGN3	244	408
IRF1	244	325-644
JUN	153-267	0
JUNB	358	0
JUND	859-1000	265-381
KDM5B	0	679
MAX	583 - 598	173-301
MXI1	0	0
MYC	151-328	125-573
NR2F2	272	0
p300	376-1000	0
PHF8	0	409
POLR2A	217-281	179-467
RAD21	137	483-1000
RBBP5	253	0
RCOR1	200-844	354
REST	235	0
SAP30	0	0

SIN3AK20	0	249
SIRT6	185	0
SMARCB1	0	156
SMC3	0	630
SP1	150	134
SPI1	0	0
SRF	0	113
STAT1	141-326	0
STAT2	216-559	0
STAT5A	333	0
TAF1	131	463
TAL1	183	0
TBL1XR1	567	166-299
TBP	219	135 - 305
TEAD4	684	0
TRIM28	385	0
UBTF	249	273-311
USF1	243	0
YY1	0	195-199
ZBTB7A	194	0
ZNF143	0	473
ZNF263	145	0
